# Safety and Efficacy Analysis of Radical Surgery for 403 Patients with Colon Cancer over 80 Years Old

**DOI:** 10.7150/jca.94016

**Published:** 2024-03-25

**Authors:** Ruilong Niu, Mandula Bao, Yujuan Jiang, Jinjie Zhang, Xiaowei Qin, Kangkang Zhang, Jianjun Bi, Wei Xing, Wei Guo, Jianwei Liang

**Affiliations:** 1 Department of Gastrointestinal Surgery, Affiliated Heji Hospital of Changzhi Medical College, Changzhi, Shanxi, China.; 2 Department of Colorectal Surgery, National Cancer Center/National Clinical Research Center for Cancer/Cancer Hospital, Chinese Academy of Medical Sciences and Peking Union Medical College, Beijing, China.; 3 Department of General Surgery, Hebei Province Hospital of Chinese Medicine; Affiliated Hospital of Hebei University of Chinese Medicine, Shijiazhuang, China.

**Keywords:** elderly, colon cancer, postoperative complications, safety, prognosis

## Abstract

**Aim:** To investigate the safety and efficacy of radical surgery in colon cancer patients over 80 years old.

**Methods:** Data from colon cancer patients aged ≥80 years who underwent radical surgery at the Cancer Hospital of the Chinese Academy of Medical Sciences and affiliated Heji Hospital of Changzhi Medical College from January 2011 to December 2022 were retrospectively analysed. Data on clinical characteristics, pathological features, perioperative data, and long-term prognosis were collected. Severe complications were classified as grade III-V. Logistic regression models were used to identify the risk factors for severe postoperative complications, and a Cox regression model was used to determine prognostic variables.

**Results:** A total of 403 eligible patients were included in the study. A total of 118 (29.3%) patients developed postoperative complications, of which 51 (12.7%) experienced grade 3-5 severe complications. Two (0.5%) patients died of pulmonary embolism and myocardial infarction during the perioperative period. The multivariate logistic regression analysis showed that preoperative albumin levels <35 g/L and right colon cancer were independent risk factors for grade 3-5 postoperative complications. In terms of prognosis, multivariate analysis revealed that overall survival was significantly affected by TNM stage III and grade 3-4 postoperative complications. In addition, TNM stage III and perineural invasion were the independent prognostic factors for disease-free survival.

**Conclusion:** Radical surgery can be performed safely in elderly colon cancer patients aged over 80 years, with an acceptable morbidity and mortality. Patients with preoperative albumin levels <35 g/L or tumors in the right colon should be alerted to the development of severe postoperative complications. In addition, the occurrence of severe complications can significantly affect the prognosis of elderly colon cancer patients.

## Introduction

Colon cancer (CC) is the third leading cause of cancer-related death in adult patients 1. With the increase in life expectancy, the proportion of elderly colorectal cancer patients is increasing, and advanced age is a recognized risk factor for the incidence of colorectal cancer 2. Colorectal cancer presents at a median age of 66 and patients older than 80 years old constitute a significant percentage of geriatric colorectal cancer patients. The older age group is associated with greater challenges in treatment because of comorbidities and frailty, which become more frequent in the older population 3. Radical resection remains the first potential treatment option for colorectal cancer. Elderly patients tend to have a complex variety of underlying diseases before surgery, the incidence of cardiovascular and respiratory complications in the perioperative period is relatively high, and surgical tolerance is low. Once serious complications occur, they will affect the quality of life and prognosis and even cause death 4. However, the perioperative safety of radical colorectal surgery remains controversial in patients aged ≥ 80 years. Meanwhile, it is also necessary to analyse the prognosis of survival after radical surgery in elderly colorectal cancer patients. Due to the various embryological origins and surrounding anatomical locations, there are differences in the molecular biology features, and treatment strategies of colon and rectal cancer patients 5. Therefore, the present study investigated the safety and survival prognosis of radical surgery in elderly colon cancer patients over 80 years old.

## Method and Materials

### Patients

**Figure 1:**
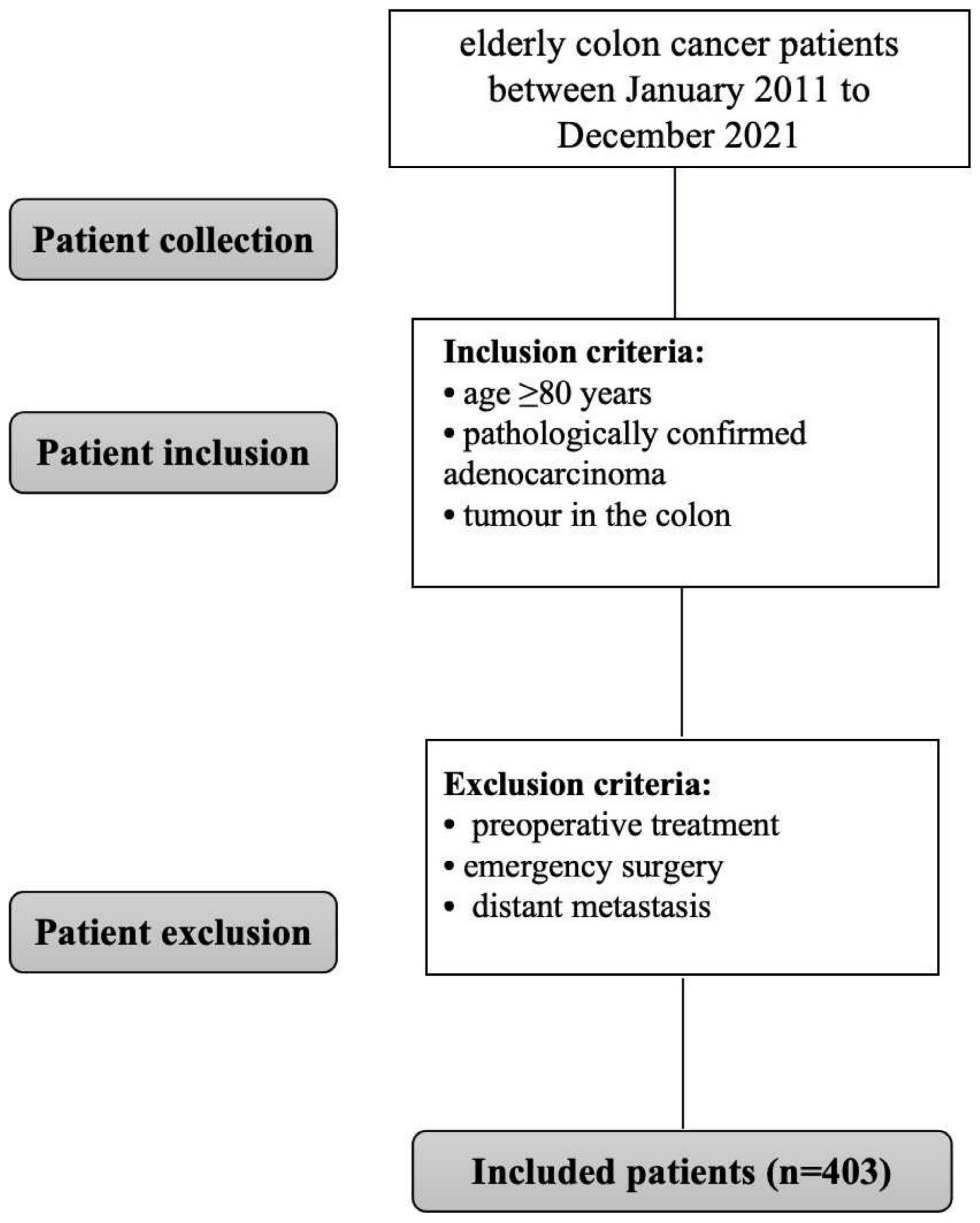
Flow chart.

The clinicopathological data of elderly colon cancer patients who underwent radical surgery at the Cancer Hospital of the Chinese Academy of Medical Sciences and affiliated Heji Hospital of Changzhi Medical College from January 2011 to December 2022 were retrospectively collected and analysed. The inclusion criteria were as follows: (1) age ≥80 years; (2) pathologically confirmed adenocarcinoma; and (3) tumour in the colon. The exclusion criteria were as follows: (1) preoperative treatment; (2) distant metastasis; or (3) emergency surgery. The development and implementation of this study was approved by the Ethics Committee, and all enrolled patients signed informed consent. The study was conducted per STARD reporting guidelines. All the procedures followed the ethical standards of the World Medical Association Declaration of Helsinki. The Institutional Review Board Committee of the institutions approved this study (LA2016-22-01). The flowchart was showed in** Figure 1.**

### Diagnosis and treatment

All patients were required to complete a cardiogram and an echocardiogram, undergo an examination of pulmonary function to assess cardiopulmonary function, and undergo an evaluation of tumour status by colonoscopy and chest, abdominal and pelvic CT. TNM staging was performed using the American Joint Committee on Cancer staging system (Edition 8). Based on differences in embryonic origin, colon cancer was divided into right colon cancer and left colon cancer. Caecum cancer, ascending colon cancer, and hepatic flexure of colon cancer were classified as right colon cancer; splenic flexure of colon cancer, descending colon cancer, and sigmoid colon cancer were classified as left colon cancer. In this study, clinicopathologic data were collected from electronic records and included age, sex, body mass index (BMI), preoperative haemoglobin level, preoperative albumin level, American Society of Anaesthesiologists (ASA) score, comorbidities, previous abdominal surgery, tumour location, tumour differentiation, tumour node metastasis (TNM) stage, blood vessel invasion, and perineural invasion. In addition, data on the perioperative outcomes, including the surgical approach, duration of operation, estimated blood loss, postoperative complications, mortality, intensive care unit (ICU) stay and postoperative hospital stay, were collected. All postoperative complications were graded according to the Clavien‒Dindo surgical grading system, with grade 3-5 defined as severe complications 6. According to the origin of complications, complications can be divided into cardiac disorders, respiratory disorders, gastrointestinal disorders, renal and urinary disorders, and other disorders. None of the patients were treated with adjuvant chemotherapy, regardless of the pathological TNM stage.

### Follow-up

Outpatient follow-up is performed every three months for the first three years after surgery. All patients received physical and laboratory examinations, including tumour markers (CEA and CA-199) at each visit, CT scans of the chest, abdomen, and pelvis every half year, and colonoscopy each year. Three years after surgery, all patients were followed up every 6-12 months until death or until December 2022. The endpoints of this study is overall survival (OS) and diseases-free survival (DFS).

### Statistical analysis

SPSS 24.0 software for Windows (IBM Corp, Armonk, NY, USA) was used to statistically analyse the data. All variables are dichotomized according to normal thresholds or mean values. Univariate analysis was performed using the chi-square test or Fisher's exact test for comparison. The variables with *P*<0.05 in univariate analysis were incorporated into logistic regression for multivariate analysis. With regard to survival analysis, the statistically significant prognostic variables in univariate analysis were subsequently tested by multivariate analysis through a Cox regression model, and the effect of each variable was assessed by the hazard ratio (HR) and 95% confidence interval (95% CI). A *P* value less than 0.05 was considered to indicate statistical significance in the present study.

## Results

### Clinical and pathological characteristics

The clinical and pathological characteristics are listed in** Table 1**. A total of 403 elderly patients aged ≥ 80 years who underwent radical colon cancer surgery were included in the study, including 240 (59.6%) men, with an average age of 82.3 years. The average preoperative haemoglobin level and albumin levels were 117.7 g/L and 37.6 g/L, respectively. There were 171 (42.4%) patients with ASA scores of grade III-IV, and 273 (67.7%) patients had preoperative comorbidities. The primary tumour was in the right colon in 179 (44.4%) patients and in the left colon in 224 (55.6%) patients. The patients had mainly moderately differentiated adenocarcinoma (69.0%), with 186 (46.2%) patients in stage II and 177 (43.9%) patients in stage III.

### Perioperative data and postoperative complications

The surgical approach was mainly laparoscopic (75.7%), and the average operation time and estimated blood loss were 161.1 min and 48.3 ml, respectively. Two (0.5%) patients died of pulmonary embolism and myocardial infarction during the perioperative period (**Table 2**). A total of 118 (29.3%) patients developed postoperative complications, of which 51 (12.7%) experienced grade 3-5 severe complications (**Table 3**). In terms of overall complications, the most common complications were wound infection (5.5%) and ileus (5.5%), followed by urinary retention (5.0%) and gastroparesis (4.7%). With regard to severe postoperative complications, the most common complications were anastomotic leakage (2.5%) and pneumonia (2.5%), followed by abdominal abscess (2.2%) and pleural effusion (2.0%).

### Univariate and multivariate analyses of grade 3-5 postoperative complications

The results of the univariate and multivariate analyses of grade 3-5 postoperative complications are summarized in **Table 4** and** Table 5**. There was a significant association between the risk of grade 3-5 postoperative complications and age (*P*=0.023), ASA score (*P*=0.001), preoperative albumin levels (*P*<0.001), and tumour location (*P*=0.002). Variables with a* P* <0.05 in the univariate analysis were further included in the multivariate logistic regression analysis, and the results showed that preoperative albumin levels <35 g/L (OR = 2.45, 95% CI=1.10-5.87, *P*=0.041) and right colon cancer (OR = 2.98, 95% CI=1.13-6.62, *P*=0.022) were independent risk factors for grade 3-5 postoperative complications.

### Survival analysis

The mean follow-up period was 57.4 months. During the whole follow-up period, 102 of the 403 patients died (25.3%), and 64 of the 403 patients had local recurrence or distant metastasis (15.9%). The univariate and multivariate analyses for OS are presented in **Table 6**. In the univariate analysis, TNM stage, perineural invasion, and grade 3-4 postoperative complications significantly affected OS (*P*<0.05). According to the multivariate analysis, OS was significantly affected by TNM stage III (HR: 5.93; 95% CI, 2.16-10.59; *P*=0.002) and grade 3-4 postoperative complications (HR: 2.57; 95% CI, 1.35-4.21; *P*=0.041). In terms of DFS, the univariate analysis showed that TNM stage, blood vessel invasion and perineural invasion significantly affected DFS. Multivariate analysis showed that TNM stage III (HR: 11.31; 95% CI, 3.42-15.30; *P*<0.001) and perineural invasion (HR: 2.30; 95% CI, 1.11-4.52; *P*=0.004) were the independent prognostic factors for DFS.

**Figure 2:**
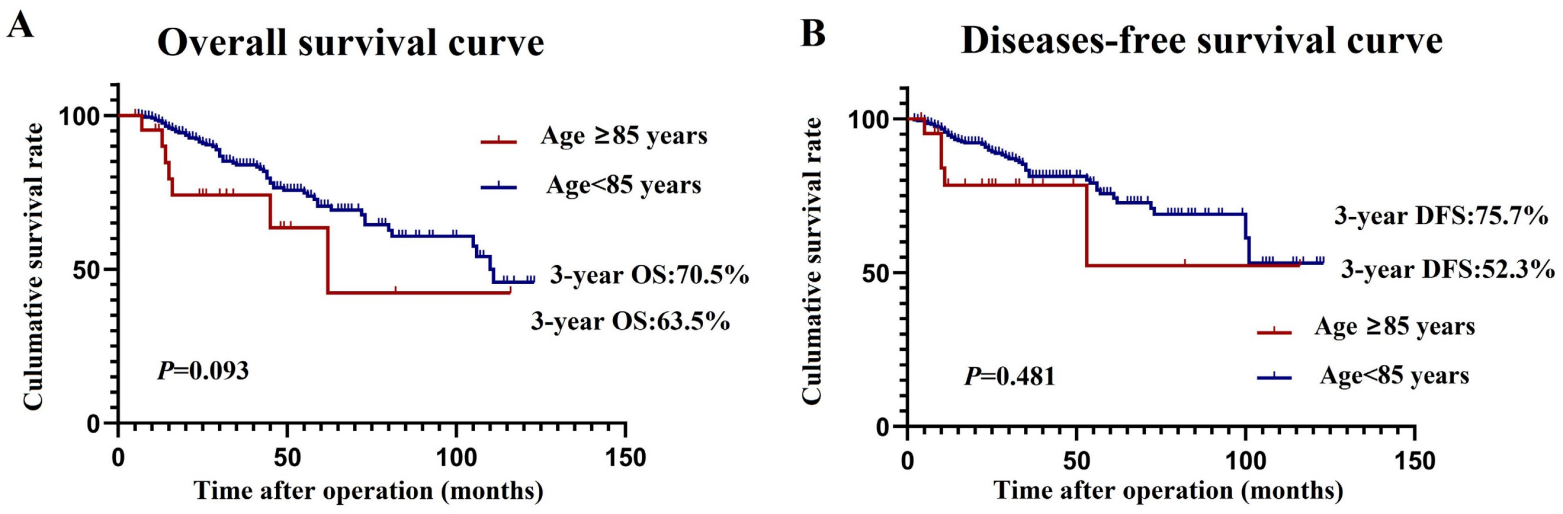
Prognosis for patients over 85 years of age and for patients aged 80 to 85 years. A, overall survival curve; B,disease-free survival curve

In addition, we performed subgroup analyses comparing prognostic differences between elder patients over 85 years of age and those aged 80 to 85 years, and the results reveal that there was no statistically significant difference between the two groups in 5-year OS (70.5% vs. 63.5%, *P*=0.093) and DFS (75.7% vs. 52.3%, *P*=0.481) (**Figure 2A, 2B**).

## Discussion

Colorectal cancer is a common malignant tumour of the digestive tract in elderly patients and seriously endangers their lives and health. With improvements in surgical technology and the continuous updating of medical equipment, more active surgical treatment strategies can be adopted for elderly patients with various organic diseases 7-9.

However, relevant studies have shown that the incidence of postoperative complications for colorectal cancer patients aged over 80 years after radical surgery is as high as 21%~31.1%, and the perioperative mortality rate is 1.1%~2.4^10^, ^11^. Therefore, it is necessary for surgeons to control postoperative complications in elderly patients with colorectal cancer. The incidence of overall postoperative complications for elderly colon cancer patients included in this study was 29.3%, and the incidence of severe complications was 12.7%, which was basically consistent with previous literature. In addition, the perioperative mortality rate calculated by this study is only 0.5%, which is lower than that previously reported in the literature. On the one hand, this may be because only deaths in the hospital were counted in this study; on the other hand, only 8 patients with preoperative ASA of grade IV were included. Conservative treatment, such as endoscopic stenting or adjuvant chemotherapy, is generally recommended for elderly colon cancer patients with severe organic diseases.

Elderly patients often have multiple underlying diseases, poor organ reserve, and a high risk of anaesthesia. Univariate analysis of this study showed that ASA score (*P*=0.001) and age ≥85 years (*P*=0.023) were associated with the occurrence of severe complications after radical colon cancer surgery in elderly patients. The reason for the increased incidence of postoperative severe complications in elderly colon cancer patients with underlying diseases is that, on the one hand, elderly patients' cardiopulmonary diseases are further aggravated by inducement, such as the rise of the diaphragm during surgery, stress inflammation, and blood loss. On the other hand, many studies have confirmed that coronary heart disease, diabetes and other underlying diseases are closely related to the occurrence of complications such as anastomotic leakage and wound infection 12-14. In addition, patients with malignant tumours have poor nutritional status and are immunocompromised 15. The results of the multivariate analysis in this study showed that preoperative albumin <35 g/L was an independent risk factor for severe complications after radical surgery in elderly colon cancer patients (OR=2.45, 95% CI: 1.10-5.87, *P*=0.041). Therefore, adequate preoperative nutritional assessment and combined enteral and parenteral nutritional support are essential. After gastrointestinal function is restored after surgery, enteral nutrition should be started as soon as possible, and amino acid or short peptide preparations can be preferred to reduce the impact on anastomotic healing.

In addition, this study found that the incidence of grade 3-5 complications after radical resection of right colon cancer was significantly higher than that in patients with left colon cancer (18.4% vs. 8.0%, *P*=0.002). Multivariate logistic regression analysis showed that right colon cancer (OR = 2.98, 95% CI=1.13-6.62, *P*=0.022) was an independent risk factor for grade 3-5 postoperative complications. Similarly, An Qi et al. analysed the postoperative complications of elderly colon cancer patients over 65 years old, and the results showed that the incidence of postoperative complications in patients with right colon cancer was significantly higher than that in the left colon cancer group (33.1% vs. 21.7%, *P*=0.015), with the average hospital stay of the former group being significantly longer than that of the latter (13.6 d vs. 10.7 d, *P*=0.018). This may be because right colon cancer is often complicated by anaemia, hypoproteinaemia and poor systemic nutritional status in elderly patients, and postoperative complications, such as poor wound healing and anastomotic leakage, are more likely to develop.

At present, the mainstream view is that the prognosis of right colon cancer is significantly worse than that of left colon cancer. Huang et al. analysed the prognosis of 1095 colon cancer patients, and the results showed that the five-year overall survival rate (47% vs. 59%, *P*=0.021) and cancer-specific survival rate (42% vs. 55%, *P*=0.023) of right colon cancer were significantly inferior to those of left colon cancer 16. In this study, the prognosis of elderly patients with colon cancer after radical surgery was analysed, and the results showed that the different tumour locations had no significant effect on the prognosis of colon cancer. Elderly patients have slower metabolism, lower tumour activity and low tumour aggressiveness, all of which lead to no significant difference in the prognosis of left and right colon cancer in elderly patients. In this study, multivariate analysis found that TNM stage III (HR: 5.93; 95% CI, 2.16-10.59; *P*=0.002) and grade 3-4 postoperative complications (HR: 2.57; 95% CI, 1.35-4.21; *P*=0.041) significantly affected the prognosis of elderly patients with colon cancer after radical surgery. The occurrence of serious complications often requires surgical intervention or reoperation. Damage to the immune system and the lengthy treatment and recovery process will certainly affect the psychological and physical health of elderly patients 17, 18. On the one hand, it will increase the possibility of tumour recurrence and metastasis; on the other hand, the deterioration of the general condition will increase the possibility of elderly patients dying from nonneoplastic diseases. In the present study, the common severe complications were anastomotic leakage (2.5%) and pneumonia (2.5%), followed by abdominal abscess (2.2%) and pleural effusion (2.0%). Meanwhile, we also have to discuss and summarize the risk factors for severe complications. Therefore, precautions should be taken to prevent such complications.

The present study had several limitations. First, there is some selection bias due to the retrospective nature of the study. Second, the study spans more than 12 years, and many therapeutic strategies and equipment are constantly being updated and developed, which may affect the results. Third, the colon and rectum have different embryological origins and surrounding anatomical locations. Elderly patients with rectal cancer were excluded from this study, so the safety and efficacy of radical surgery in elderly patients with rectal cancer still need to be further explored in the future.

## Conclusion

Radical surgery is safe and feasible for patients over 80 years of age with colon cancer, and preoperative albumin<35 g/L and right colon cancer are independent risk factors for the development of severe postoperative complications after surgery. In addition, the occurrence of severe complications can significantly affect the prognosis of elderly colon cancer patients.

## Figures and Tables

**Table 1:** The clinical and pathological characteristics of 403 enrolled patients

Characteristics	N=403
Age (years, mean±SD)	82.3 ± 1.9
Sex	
Male	240 (59.6)
Female	163 (40.4)
Body mass index (kg/m^2^,mean±SD)	24.1 ± 3.4
Preoperative haemoglobin level (g/L,mean±SD)	117.7 ± 21.2
Preoperative albumin levels (g/L,mean±SD)	37.6 ± 4.6
ASA score	
I-II	232 (57.6)
III-IV	171 (42.4)
Comorbidities (%)	273 (67.7)
Diabetes	55 (13.6)
Hypertension	141 (35.0)
Coronary heart disease	55 (13.6)
Chronic obstructive pulmonary disease	39 (9.7)
Arrhythmia	83 (20.6)
Cerebrovascular disease	26 (6.5)
Previous abdominal surgery	87 (21.6)
Tumour location	
Left colon	224 (55.6)
Right colon	179 (44.4)
Differentiation (%)	
Well	31 (7.7)
Moderate	278 (69.0)
Poor	94 (23.3)
TNM stage	
I	40 (9.9)
II	186 (46.2)
III	177 (43.9)
Blood vessel invasion	109 (27.0)
Perineural invasion	137 (34.0)

**Table 2:** The perioperative outcomes of 403 enrolled patients

Characteristics	N=403
Surgical approach	
Laparoscopic	305 (75.7)
Open	98 (24.3)
Operative time (min, mean±SD)	161.1 ± 33.2
Estimated blood loss (ml, mean±SD)	48.3 ± 13.1
Enterostomy (%)	16 (4.0)
Distance of distal margin from tumor (cm, mean±SD)	5.3 ± 1.4
Distance of proximal margin from tumor (cm, mean±SD)	11.4 ± 2.7
Postoperative complications (grade 1-5)	118 (29.3)
Postoperative complications (grade 3-5)	51 (12.7)
ICU admissions (%)	88 (21.8)
Time to first postoperative exhaust (days, mean±SD)	3.3 ± 1.5
Time to first postoperative activity (days, mean±SD)	2.5 ± 1.8
Mortality within 30 day after surgery	2 (0.5)
Postoperative hospital stay (days, mean±SD)	10.3 ± 2.2

**Table 3:** Analysis of postoperative complications in 403 patients

Complications	Grade 1-2 complications		Grade 3-5 complications		All complications	
	n	%	n	%	n	%
Total	67	16.6	51	12.7	118	29.3
Cardiac disorders						
Arrhythmia	8	2.0	6	1.5	14	3.5
Cardiac failure	4	1.0	5	1.2	9	2.2
Acute coronary syndrome	0	0	3	0.7	3	0.7
Pulmonary embolism	0	0	2	0.5	2	0.5
Respiratory disorder						
Pneumonia	6	1.5	10	2.5	16	4.0
Pleural effusion	4	1.0	8	.2.0	12	3.0
Atelectasis	3	0.7	6	1.5	9	2.2
Gastrointestinal disorders						
Anastomotic leakage	8	2.0	10	2.5	18	4.5
Ileus	15	3.7	7	1.7	22	5.5
Gastrointestinal haemorrhage	10	2.5	4	1.0	14	3.5
Gastroparesis	14	3.5	5	1.2	19	4.7
Renal and urinary disorders						
Urinary infection	5	1.2	0	0	5	1.2
Renal failure	0	0	2	0.5	2	0.5
Urinary retention	18	4.5	2	0.5	20	5.0
Other disorders						
Abdominal abscess	2	0.5	9	2.2	11	2.7
Intra-abdominal haemorrhage	8	2.0	2	0.5	10	2.5
Wound infection	19	4.7	3	0.7	22	5.5
Cerebral infarction	1	0.2	2	0.5	3	0.7
Delirium	12	3.0	2	0.5	14	3.5

**Table 4:** Univariate analyses of grade 3-5 postoperative complications

Characteristics	Total (n=403)	Grade 3-5 complications (n=51)	*P*
Sex			0.848
Male	240	31 (60.8)	
Female	163	20 (39.2)	
Age			0.023
80~85 years	355	40 (78.4)	
≥85 years	48	11 (21.6)	
ASA score			0.001
I-II	232	18 (35.3)	
III-IV	171	33 (64.7)	
Body mass index			0.796
<24 kg/m^2^	228	28 (54.9)	
≥24 kg/m^2^	175	23 (45.1)	
Preoperative albumin levels			<0.001
<35 g/L	69	18 (35.3)	
≥35 g/L	334	33 (64.7)	
Preoperative hemoglobin level			0.115
<120 g/L	157	25 (49.0)	
≥120 g/L	246	26 (51.0)	
Comorbidities			0.081
Yes	273	40 (78.4)	
No	130	11 (21.6)	
Previous abdominal surgery			0.469
Yes	87	13 (25.5)	
No	316	38 (74.5)	
Surgical approach			0.209
Laparoscopic	305	35 (68.6)	
Open	98	16 (31.4)	
Date of surgery			0.205
Before January 1, 2014	105	17 (33.3)	
After January 1, 2014	298	34 (66.7)	
Operative time			0.875
<160 min	249	31 (60.8)	
≥160 min	154	20 (39.2)	
Estimated blood loss			0.085
<50 ml	279	30 (58.8)	
≥50 ml	124	21 (41.2)	
Tumour locations			0.002
Left colon	224	18 (35.3)	
Right colon	179	33 (64.7)	
TNM stage			0.305
I-II	226	32 (62.7)	
III	177	19 (37.3)	

**Table 5:** Multivariate analyses of grade 3-5 postoperative complications

Characteristics	Multivariate analysis		
	*OR*	95%*CI*	*P*
ASA score: III-IV	2.02	0.93~4.71	0.674
Age: ≥85 years	4.43	1.73~11.52	0.083
Preoperative albumin levels:<35 g/L	2.45	1.10~5.87	0.041
Tumour locations: right colon	2.98	1.13~6.62	0.022

**Table 6:** Univariate and multivariate analysis for overall survival and disease-free survival

Variables	Overall survival				Diseases-free survival			
	Univariate analysis		Multivariate analysis		Univariate analysis		Multivariate analysis	
	HR (95%CI)	*P*	HR (95%CI)	*P*	HR (95%CI)	*P*	HR (95%CI)	*P*
Sex: male/female	1.12 (0.61-2.09)	0.719			1.75 (0.58-2.35)	0.620		
Age: ≥85/<85years	2.33 (0.91-5.92)	0.093			1.50 (0.71-6.37)	0.481		
ASA: III-IV/I-II	1.33 (0.92-2.34)	0.211			1.24 (0.79-2.52)	0.492		
Comorbidity: yes/no	1.12 (0.78-2.53)	0.562			0.92 (0.88-3.20)	0.892		
Preoperative albumin levels: ≥35/<35 g/L	1.43 (0.91-4.95)	0.256			1.20 (0.71-4.20)	0.491		
Preoperative hemoglobin level: ≥120/<120 g/L	1.44 (0.82-5.92)	0.623			1.31 (0.77-5.42)	0.521		
Surgical approach: open/laparoscopic	1.31 (0.77-2.16)	0.253			1.63 (0.53-7.33)	0.592		
Tumour location: right colon/left colon	3.42 (0.94-9.32)	0.111			4.30 (0.91-11.42)	0.081		
TNM stage								
I	Reference	-	Reference	-	Reference	-	Reference	-
II	3.60 (0.92-8.30)	0.103	2.36 (0.77-5.52)	0.324	4.20 (2.92-7.30)	0.039	3.42 (0.72-7.51)	0.152
III	14.37(6.11-33.82)	<0.001	5.93 (2.16-10.59)	0.002	23.37(8.94-63.52)	<0.001	11.31 (3.42-15.30)	<0.001
Differentiation								
Poor	Reference	-			Reference	-		
Median	0.63 (0.33-1.26)	0.188			0.64 (0.49-1.75)	0.142		
Well	0.72 (0.41-1.54)	0.273			0.61 (0.37-1.15)	0.092		
Blood vessel invasion:yes/no	2.55 (0.94-5.91)	0.131			4.22 (1.80-7.31)	0.037	2.81 (0.90-8.21)	0.081
Perineural invasion:yes/no	3.24(2.21-5.14)	0.022	1.63 (0.81-3.47)	0.175	4.31 (2.33-11.34)	<0.001	2.30 (1.11-4.52)	0.004
Retrieved lymph node	0.82 (0.70-1.73)	0.523			0.77 (0.52-2.70)	0.590		
Postoperative complication	1.36(0.84-4.98)	0.412			1.52 (0.71-6.33)	0.629		
Postoperative complication (grade 3-4)	2.32 (1.41-5.14)	0.033	2.57 (1.35-4.21)	0.041	2.74 (0.92-5.90)	0.080		

## Abbreviations

CC: Colon cancer
BMI: body mass index
ASA: Anaesthesiologists
TNM: tumour node metastasis
ICU: intensive care unit
DFS: diseases-free survival
OS: overall survival
HR: hazard ratio
CI: confidence interval
